# Heredity

**Published:** 1886-03

**Authors:** S. D. Robertson

**Affiliations:** Chicago


### THE DENTAL REGISTER.

VOL. XL.] MARCH, 1886. [No. 3.

### COMMUNICATIONS.

### Heredity.

BY S. D. ROBERTSON, D.D.S., CHICAGO.

Since that divine proclamation was uttered, no other fact in history has been more positively verified nor more generally conceded by all classes regardless of religious belief, than that the "iniquities of the fathers are visited upon the children unto the third and fourth generations."

The term "heredity" implies certain physical or mental qualities transmissible from parent to offspring, or, "heredity is that biological law by which all beings endowed with life tend to repeat themselves in their descendants; it is for the species what personal identity is for the individual."

In starting out upon a subject so broad and deep a subject embracing not only the preservation of material or physical form, whether of the animal or vegetable kingdom, but the preservation of function with which all organisms are endowed, the aim will be to avoid hypotheses, in which the volumes upon heredity so richly abound, and dwell upon facts of common observation. That "like begets like," is, in the main true—as true to-day as it was six thousand years ago, but, to say "like by like" with mathematical precision is to interfere with the latitude granted this law, in which may obtain improvement or degeneration.

While ignorance is said to be bliss under certain circumstances, to be ignorant upon a subject of such vast import as this, affecting the health, lives, and happiness of not only those who now live, but, reaching forward into the future of unborn millions, how great must be the responsibility and accountability. Those who have never informed themselves upon hereditary transmission will marvel when they comprehend the wonderful features and divers phases it presents. If men would give as much attention to the laws of hereditary transmission in their own race, as they bestow upon the propagation of many of the domestic animals the human family would be immeasuredly advanced intellectually, physically, and morally. That individuals and species which have advantages in vigor, agility or better adaptation to surrounding circumstances, will live and reproduce themselves, while those less favored will perish, is universally known. This sorting process Darwin calls, "Natural Selection," Huxley, "The Survival of the Fittest."

The relative influence which each parent exerts over their progeny as to the qualities transmitted to them is not definitely known. The grand structure of the body, the degree of development of the bones and muscles, the tendency to obesity or the opposite, seem to depend as frequently upon one parent as the other in the human family; but in the brute creation—the horse for example—the male most frequently determines the general form and size of the body.

However plausible the doctrine of crossing the races may have been, it is now pretty generally conceded that the highest degree of vigor may be attained by the concentration of the energetic races. It is proven that the blood of all races can not be blended with impunity—as degradation will follow. There must be a strong sympathy in order to be successful though seemingly opposed.

The progeny of the strong and perfect resulting from a union with the weak and inferior will be but of an ordinary quality; while that resulting from a union of strength and perfection on both sides, will be a concentration of vigor and genius. This principle is understood and practiced in breeding and rearing horses. The famous race horse Eclipse, was produced by a successive union of the accumulated blood of a pure strain unimpared by combining. Horsemen will advocate that they wish to concentrate a certain strain or pedigree that will predominate and give to the horse those qualities which they desire. Hence you will hear them say, "My horse is bred so and so, sired by such a horse, his dam is lady so and so, she by so and so, 2d dam the famous trotter so and so, she by so and so, he by so and so, and *he* by imported messenger 3rd dam thorough hilbred.

There is once in a great while an individual who takes pride in tracing back and keeping a correct record of his or her family lineage. I know of one such person who can give you not only the original stock she descended from, but also the Christian names of each succeeding generation from the landing of the Pilgrim fathers down to the present time.

By referring to the family record in the old family bible, some of us may learn the names of our grandparents on either side but as a rule what do we know or care about the qualities or pedigree back of them, unless for the purpose of proving some title of original inheritance, then we are anxious to know *all* about it. I can conceive of cases, however, wherein it would be an advantage to the progeny to know as little of the qualifications of their ancestors as possible and *vice versa*. Sometimes the complexion of the two parents appears unattenuated in the offspring, but from observation it is believed that in the progeny resulting from a union of the ebony and white races the color of the father predominates over that of the mother.

A very curious and noticeable fact pertaining to this subject, is the transmission of special marks or deformities exhibited by one of the parents, or more remote ancestors not common to the species, which have a tendency to show themselves in alternate generations, or even at greater intervals. I refer to moles, harelip, growths of hair in unusual places, an additional number of fingers or toes. Special malformations of the heart and other organs have been traced to hereditary influence.

Hereditary tendency to mental diseases is eminently propagable, chief among these are hallucination, monomania, suicide, mania, dementia, and idiocy. In France one case in every three. Among the peasants one in every ten cases of insanity from all causes is found to occur in families predisposed to mental alienation. In Italy the proportion is nearly the same, individuals who have inherited an unhealthy cerebral organization or physical diseases, fall victims to insanity more frequently than those born physically and mentally robust.

Abercrombie cites a case of hereditory hallucination where the reason remained intact, although hallucination is usually coupled with insanity. This disposition is of such a nature that if the patient meets a friend in the street, he can not tell at once whether it is an actual person or a phantasm ; by dint of attention, however, he can make out a difference between the two, usually he corrects the visual impressions by touch or by listening for the foot-falls. His father and grandfather were like him.

This man was in the flower of his age, of sound mind, in good health and engaged in business. A man in the Lyons hospital was subject simultaneously to hallucinations of taste and smell, tormented by disgusting odors and taste, he spent whole hours blowing his nose and spitting. His father died in the same hospital from the effect of mania with hallucination. A thoughtful professional man of mature age, of regular habits, having no strong passions, and beyond the reach of want, committed suicide on the 17th of October, 1769, leaving behind him, addressed to the council of his native city an apology for his voluntary death, which it was not thought advisable to publish, lest men should be encouraged to quit a life whereof so much evil is spoken. His father had committed suicide at the same age as himself.

What hidden disposition of mind, what sympathy, what concurrence of physical laws caused this father and his two sons to perish by their own hands and by the same form of death, just when they had attained the same age?

So of helancholia, or mypochondria—called by some authors Lypemania. A woman effected with Lypemania was sent at the age of 42 to an asylum, and there died. It was discovered that her grandfather and her mother had been insane, and her son, barely 15 years of age already gave signs of Lypemania. In 482 cases of this disorder, there were found to be hereditary, 110. Particular forms of degeneration are propagated in some families, so that we see the suicidal impulse in one, while the uncontrollable and insatiable desire for alcoholic stimulents is the heritage of another.

The passion known as dipsomania, or alcoholism, is so frequently transmitted that all are agreed in considering its heredity—as the rule, not, however, that the passion for drink is always transmitted in that identical form, for it often degenerates into mania, idiocy, and hallucinstion, conversely insanity in the parents may become alcoholism in the descendents. This continual metamorphosis plainly shows how near passion comes to insanity, how closely the successive generations are connected, and consequently, what a weight of responsibility rests upon each individual. A frequent effect of alcoholism is a partial atrophy of the brain, *i. e.*, a wasting away—a shrinking down, a lack of nourishment, the organ is reduced in volume so that it no longer fills the bony cavity or cranium, the consequence is a mental degeneration, resulting in a progeny lunatics and idiots. Gall speaks of a Russian family in which the father and grandfather had died prematurely, the victims of this taste for strong drink. The grandson, at the age of 5, manifested the same liking in the highest degree. A French author gives the names of several families in which the taste for strong drink was transmitted by the mother. Dr. Morel had again an opportunity of proving the hereditary effects of alcoholism in the children of the "Commune;" he inquired into the mental state of 150 children, ranging in age from ten to seventeen years, most of whom had been taken with arms in their hands behind the barricades. This examination he says, has confirmed me in my previous convictions as to the painful effect produced by alcohol, not only in the individuals who use this detestable drink to excess, but also in their descendents.

On their depraved physiognomy is impressed the threefold stamp of physical, intellectual, and moral degeneracy. Dr. Anstie, of London, England, in an article on Alcoholism, after referring to various exciting and predisposing causes of this disease, says: There is another kind of predisposing cause which is constant in its operation, and which is probably, *at least*, as influential, both in producing alcoholic excess and in aggravating its ill-effects as any other, viz., a peculiar inherited constitution of the nervous system.

In the course of a large experience of alcoholism among the hospital out patients, he says he has been greatly struck with the number of drinkers who have informed him that their relatives either on the paternal or maternal side have also been given to drink; and a still larger number are found on inquiry to come of families in which some nervous disorder (especially insanity, epilipsy, and neuralgia), have been markedly prevalent.

The doctrine of the hereditary transmission of neurosis (a disease of the nervous system), which, according to the special presence of external circumstances, may take the form of intellectual insanity, of emotional impulsiveness, combined with moral weakness, or, on the other hand, of convulsive or neuralgic affections, has been much insisted upon by recent alienist writers, and especially by Morean, in his very able treatise on morbid psychology. Dr. Anstie further says, that his own experience has led him to a firm conviction that particular cases of nervous degeneration affecting individuals do very frequently lead to the transmission to the offspring of those persons, of an enfeebled nervous organization which renders them peculiarly liable to the severer neurosis, and which also makes them ready victims of the temptation to seek oblivion from their mental and bodily pains through narcotic and alcoholic indulgence.

That things often work in a vicious circle to this end cannot be denied, and that the nervous enfeeblement produced in an ancester by great excesses in drink or otherwise, is reproduced in his or her various descendants, with the effect of producing insanity in one, epilepsy in another, neuralgia in a third, and so on. Among the higher classes, where it is easier than in the case of the poor and lower, to obtain tolerably complete family histories, extending over two or three generations, careful enquiry elicits facts of this kind with surprising frequency.

The great majority of the most inveterate and hopeless cases of alcoholic excess among the higher classes, are produced by two factors, of which the least important is the circumstance of external momentary temptation in which the patient has been placed, while the more *momentous* and weighty cause is derived from an inherited nervous weakness, which renders all kinds of bodily and mental trouble especially hard to be borne. In view of this the fatal rapidity with which habits of intemperance exaggerate themselves, is only what might be expected, seeing that the nutrition of nerve centers would be still further impaired by each successive indulgence in poisonous doses of alcohol, and the power of moral resistence to feelings of depression and misery would be proportionately weakened. As regards those passions which have their origin in the desire for eating, it is impossible to cite facts to prove their heredity so remarkable. Gluttony and voracity seldom lead to such deplorable results as alcoholism. It is not, however, difficult to find families in which voracity is inherited. This has been observed in the Bourbons. Saint Simon informs us that Louis XIV. was a man of extraordinary greediness, and the same was the case with his brother. Nearly all this king's sons were gourmands and great eaters, and this passion has been transmitted to their descendants.

A more curious case, and one comparable to alcoholism, owing to its morbid character, is the fact of a hereditory tendency to cannibalism.

The German phrenologist, Gall, tells of a Scotch family possessed of an instinctive propensity to cannibalism, which persisted through several generations Sundry members of this family paid the penalty of this with their lives, and others had to be placed under surveillance.

It is probable that the children of cannibals brought up in Europe would exhibit the like tendencies in the midst of our own civilization. The influence of sex bears somewhat upon this subject. Recent researches into the statistics of different countries affirm that owing to the mental wear and tear in the competition of life, and their more frequent addiction to intemperance and other excesses, men are the more frequent victims of insanity than women, while women are more exposed by constitution to the exciting causes, and consequently, more liable to hereditary insanity. French authorities record that of 467 cases of mental affections, 279 were traceable to the mother.

When the physical or mental taint exists on the side of the mother a greater number of children are borne of unsound body or mind, manifested in various forms, such as epilepsy, mania, eccentricity, melancholia, or delusions — an account of the last sometimes being exhibited in successive generations.

While it would be grossly false to say that all the bad qualities exhibited in human depravity were inherited from the mother, we have a right to assume that those grand and noble characteristics which we see and admire in true men and true women are *not* inherited from a bad mother.

One writer has said all nature is infinitely sacred, while mother is earth's holiest shrine. Who that lives but owes a debt of eternal gratitude to mother for what she has done for us, nurturing us through our germinal period, giving us an existence, caring for us in infancy, watching over us in childhood, and bestowing upon us her vigilant counsels and admonitions, even up to manhood—determining the destinies not only of individuals, but of communities, nations, and kingdoms.

Hence every mother is entitled to whatever of filial affections and devotion it is in our power to bestow. They are heathenish who neglect her, even though she may abuse them. Let all do our utmost for her, administer to her every comfort, for in doing this we shall only return a small remuneration in comparison for what she has done for us. Parents frequently live over again in their offspring, for children usually resemble their parents, not only in countenance and bodily conformity, but also in the general characteristics of their minds, in both virtues and vices.

The Edinburgh phrenologist and philosopher, Mr. Combe, remarks: That those who desire bodily and mental soundness in their offspring, ought carefully to avoid intermarrying with individuals who are either feeble in constitution, or strongly predisposed to any serious disease, and above all, the greatest care should be taken against the union of the same morbid predisposition in both father and mother.

The child receives its organization either good or bad, physical or mental from its parents. It is well known that a father whose system has become debilitated or impressed by any disease, communicates a similar affection to his offspring. So also does the mother transmit her infirmities in the same manner but in a more marked degree. The most prominent of the diseases which are transmissible are pulmonary consumption, scrofula, syphilis, dyspepsia, gout, and cancer. While various reforms are being cried by the different political parties, I believe the greatest reform that this country needs to-day is reform in the marriage laws, prohibiting the marrying of individuals affected with such hereditary diseases as I have named. Even ancient Rome was far in advance of our own civilization in this respect. If you have never had your life insured and you should make application for a policy, stating in your application that you had lost two or more near relatives with consumption, heart disease, or cancer, see how quickly your application would be rejected. Besides the direct inheritance of an infirm constitution there are many other causes which may deteriorate the race.

The union of parents too nearly allied in blood is a prominent cause as observed among some of the royal families of Europe, as also in private life. Marriage before full maturity and development, particularly in delicate females, as well as great disproportion in age between the parents are potent causes of infirm health in their children. Conditions of the mind at the time of conception, during pregnancy, and lactation, I shall try to show, have their permanent effects upon the offspring. Anxiety of mind, or unusual depression of the spirits in the father have been found imprinted in ineffaceable characters on the organization of the child, and not a few instances are known in which idiocy in the offspring has been the direct result of an accidental intoxication on the part of a generally temperate father, and if an accidental or an occasional drunk will precipitate such a legacy upon the unborn, what must be the results upon the progeny of chronic drunkards? A father sometimes grieves over, and finds fault with the follies of a wayward son without suspecting that he actually derived his origin from some forgotten irregularity of his own.

Fowler in his work on Sexual Science relates where a drunkard's wife declares that she can trace minutely in the great diversities of character and disposition in her numerous children just those very states of mind existing when she was bearing them. The first was peculiarly beautiful and amiable, but the husband began to drink, which overclouded her sky and awakened her displeasure, and the next child corresponds with this state of her mind. Then came poverty and that severe buffeting of the waves of adversity, which called out all her force, and the unamiable traits and character of the children born during this sad period corresponds with it, and so she reads in their characters the history of her own hidden life and feelings. The domestic history of all large families will be found inscribed upon the different dispositions of each as compared with the others.

Biblical history corroborates this fact, Hagar's state of mind while pregnant illustrates the truth of this natural law, she was insolent and mad because likely to bring Abraham the desired heir, so that Sarai became jealous of her and after repeated quarrels became desperate and drove Hagar into the wilderness to starve. The fruit of this conception resulted in the birth of Ishmael who was so unfortunate you remember as to hate and be hated by everybody, nor did this disposition end in the birth of Ishmael, but was transmitted to his progeny which characterized the Ishmaelites ever after.

Draw the contrast in the dispositions of Hagar and Hannah the mother of Samuel, and Mary the mother of Christ.

Is it not reasonable to suppose that Hannah's piety had much to do with Samuel's early consecration and love for the sanctuary? And Mary, the embodiment of perfection, womanly beauty and sweetness of disposition, had her robust health and happy frame of mind during Christ's nativity anything to do in the formation of one of the grandest, most noble, and sublime characters known to humanity? The ancient Greeks seem to have been familiar with the laws of heredity; pregnant females were cared for and watched over with the most sacred caution, no one was allowed to disturb or vex them in any manner, and their chambers were provided with the most beautiful specimens of sculpture and paintings, such as the figures of Apollo, Narcissus, Castor and

Pollux, that they might dwell upon their fine proportions with that complacency of spirit which beautiful objects always inspire.

For the same reason the Spartans took their wives to the battlefield that their children unborn might be influenced by the songs and triumph of victory.

Maternal irritability is the great cause of ill-natured children. That ugly boy always teasing his sisters, quarreling with his schoolmates, talking saucily to his mother and grandmother, tormenting the cat and dog, thrashing the cows around the barnyard with a cornstalk in one hand and the tail in the other and continually getting into trouble, cursing and fighting, is the more to be pitied the worse he is just as he would be had he inherited club feet, a hunch back, cancer or syphilis. Why? For the simple reason that his mother and father had stamped those irascible qualities into his disposition by their repeated quarrels during the child's nativity, and *they alone* should be held responsible.

An irritable mother completely broken down in spirit said of her daughter: "She is a perfect mule, even in trifles, sits sometimes all day absolutely refusing to do anything, not even to comb her own hair; becomes furious and remains sulky and speechless all day without any provocation, teases the life out of her little brother and when told to stop declares she has not spoken to him since morning. Often when dressed for church, tears off her clothes, dishevels her hair, heeds neither persuasion nor reason, and in all respects is the worst girl I ever saw. I could not believe it possible for so bad a girl to exist. During her fœtal life I had the worst of servants, impudent, lying, thievish, which provoked me almost to death, so that I was about crazy. Who was the responsible party in this case? Mother, daughter, or servants?"

A single member of a family is sometimes observed to be thus affected, which cannot be otherwise accounted for.

The constitutional aversion to weapons of every kind shown by James I of England is ascribed, and not without reason, to the constant anxiety and apprehension under which Mary lived during gestation.

So the philosopher Hobbes ascribed his excessive nervous sensibility and timidity to the fear in which his mother lived on account of the threatened Spanish invasion.

I have in my library a small work entitled "The Jukes," a study in crime, pauperism, disease, and heredity by R. L. Dugdale, member of the executive committee of prison association, New York.

It appears from the history of this family, that within a period of 75 years there have sprung from 5 sisters 1,200 persons. The history of 709 of the 1,200 shows the following facts:

| | |
|---|---|
| Paupers | 280 |
| Years of pauperism | 1,798 |
| Criminals | 140 |
| Years of infamy | 750 |
| Thieves | 60 |
| Murderers | 7 |
| Prostitutes | 165 |
| Illegitimate children | 91 |
| Number of persons contaminated by syphilitic disease | 480 |
| Cost to the State in various ways | $1,308,000 |

The last case in the illustration of this subject, from among many similar ones, is the following recorded by Baron Percy, an eminent French military surgeon, as having occurred after the seige of Landau, in 1793: The women of the town were kept for sometime in a constant state of alarm by a violent cannonading, when suddenly the arsenal blew up with a terrific explosion. Out of 92 children born within a few months afterwards, Percy states that sixteen died at birth, 33 languished for from 8 to 10 months and then died, 8 became idiotic, and died before the age of 5 years, and 2 were born with numerous fractures of the bones caused by the convulsive start of the mother, excited by the fright. So that 2 out of every 3 were actually killed through the medium of the mother's alarm. These instances, with much other evidence, are sufficient to establish the existence of a direct relation between the general condition of the mother, and the general constitution of the child.

By many women gestation is regarded with alarm—as a period of danger, and worse than doubtful in its results. This is not true, but it is a consolation to know that this period is not naturally fraught with danger, but is rendered perilous only, or chiefly so, by neglect or mismanagement, from the moment of conception. If there is one duty more paramount than another, it is the obligation on the part of the father and mother both, to secure for her by every possible means the highest state of mental and bodily health, of which her constitution is susceptible.

The cause of resemblance in offspring to parents and ancestors has been made a subject of careful study by scientific men.

The most recent theory adopted, and the one which presents the most plausible hypothesis, is that known as the doctrine of "Pangenesis." This is the technical name for Darwin's theory of variation in animals and plants under domestic cultivation.

It is a well known fact to physiologists that every part of the living body is made up of cellular elements which have the power to reproduce their kind in the individual, thus repairing the loss resulting from waste, be that from disease or injury. Each cell produces cells like itself. It is also known that there are formed in the body numerous central points of growth termed "nuclei." In every group of cells is formed one of these central cells (or nucleolus) from which the others originated and which determiues the form of their growth.

Dr. Kellogg cites as an illustration of this fact an experiment in which the spur of a cock was grafted on the ear of an ox, which lived and thrived in this somewhat novel situation for eight years, growing to the length of 9 inches and weighing nearly a pound. A tooth has been made to grow on a rooster's comb in a similar manner. The tail of a pig has been transplanted from its normal position to the back of the animal and retained its sensibility.

The theory of pangenesis supposes that these centres of nutrition form and throw off not only cells like themselves, but very minute granules called gemmules, each of which is capable under suitable circumstances of developing into a cell like its parent.

These minute granules are distributed throughout the system in great numbers. The essential organs of generation perform the task of collecting gemmules, and forming them into sets, each of which constitutes a reproductive element, and contains in a rudimentary form a representation of every part of the individual including the most minute peculiarities. It is supposed from this that each ovum or egg contains not only the gemmules necessary to reproduce the individuals who produce, but also a number of gemmules which have been transmitted from the individual progenitors.

If this theory be true it is easy to understand all the problems of heredity.

It will be seen then that zoosperm actually contains in an embryonic form every organ and tissue of the individual producing it; the same may also be said of the ovum.

In other words the reproductive elements are complete representatives in miniature of the parents, and contain the same peculiarities, the same eccentricities, the same diseases and natural deformities as the parents.

Various modifying circumstances are sufficient to explain the seeming and also the real dissimilarities between parents and children.

